# Atmospheric microplastic deposition in an urban environment and an evaluation of transport

**DOI:** 10.1016/j.envint.2019.105411

**Published:** 2020-03

**Authors:** S.L. Wright, J. Ulke, A. Font, K.L.A. Chan, F.J. Kelly

**Affiliations:** a MRC Centre for Environment and Health, Analytical, Environmental and Forensic Sciences, King’s College London, London, United Kingdom; b Environmental Research Group, Analytical, Environmental and Forensic Sciences, King’s College London, London, United Kingdom; c Institute of Pharmaceutical Science, School of Cancer and Pharmaceutical Sciences, King’s College London, London, United Kingdom

**Keywords:** Microplastics, Atmospheric deposition, Air pollution, Urban

## Abstract

Microplastics are a global environmental issue contaminating aquatic and terrestrial environments. They have been reported in atmospheric deposition, and indoor and outdoor air, raising concern for public health due to the potential for exposure. Moreover, the atmosphere presents a new vehicle for microplastics to enter the wider environment, yet our knowledge of the quantities, characteristics and pathways of airborne microplastics is sparse. Here we show microplastics in atmospheric deposition in a major population centre, central London. Microplastics were found in all samples, with deposition rates ranging from 575 to 1008 microplastics/m^2^/d. They were found in various shapes, of which fibrous microplastics accounted for the great majority (92%). Across all samples, 15 different petrochemical-based polymers were identified. Bivariate polar plots indicated dependency on wind, with different source areas for fibrous and non-fibrous airborne microplastics. This is the first evidence of airborne microplastics in London and confirms the need to include airborne pathways when consolidating microplastic impacts on the wider environment and human health.

## Highlights

• Microplastics were present in atmospheric deposition in central London.
• Comparing equal size classes, levels were 20 times greater than in a remote location.
• Fibrous morphologies dominated and polyacrylonitrile was the most common polymer.
• Local source areas influenced microplastic levels.

## 1 Introduction

Following current trends, global plastic production is projected to reach an accumulative 25 billion metric tonnes by 2050 (Geyer et al., 2017). Owing to its position as an integral material today is one of its key attributes; durability. Consequently, the proportion of the 4.9 billion discarded tonnes (Geyer et al., 2017) which has reached the environment, as opposed to landfill, recycling or incineration, has accumulated and persists in ecosystems worldwide. However, plastic does eventually lose mechanical integrity, via biotic and abiotic degradation pathways, including photooxidation and abrasion. Eventually, it emits particles known as microplastics to the wider environment (Klein et al., 2018). Microplastics define plastic particles ≤5 mm in size, although there are efforts to redefine them as ≤1 mm in size, as recommended by (Hartmann et al., 2019), and include primary microplastics which have been purposefully manufactured.

Microplastics have predominantly been recognised as marine contaminants (Thompson et al., 2004, Desforges et al., 2014) with estimates of 93 to 236 thousand metric tons floating on the global sea surface (van Sebille et al., 2015). However, scientific perception is changing; evidence is emerging to show microplastics also permeate freshwater (Faure et al., 2015, Morritt et al., 2014) and terrestrial environments (Liu et al., 2018, Rillig, 2012). Considering where plastic is produced, used and disposed, combined with its degradation pathways, it can be anticipated that microplastics are emitted to and occur in our immediate environment on land. Microplastics have been measured in atmospheric fallout in the megacities of Paris, France (Dris et al., 2016) and Dongguan, China (Cai et al., 2017). (Dris et al., 2016) found average deposition rates of 110 ± 96/m^2^/d and 53 ± 38/m^2^/day in total atmospheric deposition collected at an urban and suburban site in Paris, respectively. In Dongguan City, China, an average deposition rate of 36 ± 7/m^2^/d was reported (Cai et al., 2017). Recently, microplastics were measured in deposition at a remote, pristine mountain catchment (French Pyrenees) at a comparable daily rate (365/m^2^/d) (Allen et al., 2019). Air mass trajectories suggested the microplastics had transported over a distance up to 95 km, reaching the sparsely inhabited areas (Allen et al., 2019). This was further supported by observations of microplastics in remote (Arctic, Swiss Alps) and urban (Bremen, Germany) snow, although estimated annual deposition in these regions was low (average 1.4–66 microplastics/m^2^) (Bergmann et al., 2019). Atmospheric transport of urban atmospheric microplastics is unknown, but a likely pathway to pristine environments.

In the environment, microplastics can have detrimental effects on aquatic organisms when ingested (Sussarellu et al., 2016, Wright et al., 2013). The potential for human exposure via inhalation to occur is also of concern (Wright and Kelly, 2017). In addition to increasing our knowledge of long-range microplastic transport, it is equally important to examine microplastic contamination in major population centres. Here, concentrations are likely to be higher and therefore exposures greater compared to remote locations, however, there is a paucity of evidence on the extent of this phenomenon. The current study aims to contribute to the limited existing knowledge by examining whether microplastics are present in total (wet and dry) atmospheric deposition in a megacity environment, London (UK). Quantity, size, shape and polymer type are assessed, thereby improving our understanding of the characteristics of microplastic deposition in an urban environment. Local and longer-range geographical sources are evaluated for the first time using microplastic-specific characteristics (shape/aerodynamic equivalent diameter and density).

## 2 Materials and methods

The main aim of the study was to assess whether microplastics contaminate the air in central London. This was achieved via the following objectives (1) collect total deposition samples; (2) count suspected microplastics in samples; (3) confirm particle composition using FTIR; and (4) explore potential local geographical sources and transport.

### 2.1 Field sampling

Total atmospheric deposition samples were collected from a nine-story-high roof (~50 m above ground level) at a riverside urban site in Central London (51.5111° N, 0.1171° W). Outdoor total atmospheric deposition samples were collected twice a week for 4 weeks, from the 19th of January to the 16th of February 2018 using an aluminium rain gauge with a 0.03 m^2^ (200 mm diameter) orifice (NovaLynx 260–2510 Standard Rain and Snow Gauge, US) which was continuously exposed, resulting in three or four-day sampling periods. Meteorological data was collected throughout the sample period via a Campbell Scientific weather station consisting of a datalogger (CR10X), temperature and relative humidity probe (HMP155A), wind monitor (wind speed and direction; 05103-L), and a tipping bucket rain gauge (SBS500).

Three 1 L Duran bottles (with all plastic components removed) were filled with 1 L of filtered (0.45 μm) deionised (DI) water and covered with aluminium foil. Standing downwind, the contents of the 1 L bottles were carefully poured around the perimeter of the rain gauge surface, rinsing the sample into the central vessel. Each time, the sample was poured back into its original bottle, resulting in three successive washes. The aluminium foil was replaced to keep the sample covered. The samples were transported back to the laboratory, where they were kept in the dark at 4 °C until further processing (within approximately 4 days of collection).

### 2.2 Sample preparation for analysis

To concentrate samples onto a substrate, they were initially vacuum filtered onto 0.2 μm pore size alumina-based membrane filters (GE Healthcare Whatman™ Anodisc™, UK). The resulting filtered water was then re-used to rinse the sample bottle three times, filtering the contents onto the sample filter each time. Finally, the filtered water was carefully poured down the sides of the top unit, to ensure any particles stuck to the sides were collected. The filter was immediately transferred to a glass petri dish and covered with the corresponding glass lid. The petri dishes were placed in an oven at 40 °C for approximately 4 h to dry, then stored in the dark at room temperature.

To facilitate spectroscopic analysis, samples were transferred to silver membrane filters (1.2 μm pore size, Sterlitech, WA, USA), which have low signal interference compared to the alumina-based membrane filters (which were originally deemed appropriate). Samples were placed in glass beakers with 10 mL 10% HPLC-grade methanol in ultrapure H_2_O and extracted by standing beakers in a sonicating bath (Clifton™ Ultrasonic Bath, Fisher Scientific, UK) for 1 min. The methanol-particulate matter suspension was then vacuum-filtered. The original alumina filter was transferred into a new beaker and the process repeated as before. Finally, both beakers used in the sample extraction were thoroughly rinsed with 10% methanol and vacuum-filtrated onto the silver membrane filters (Sterlitech, WA, USA). Preliminary work found recovery rates of 86% (PS), 90% (PP, HDPE) and 93% (PET) for reference microplastics following this extraction procedure.

Nile Red (NR) is a lipophilic dye which has been used to stain microplastics to facilitate their identification and analysis in samples (Maes et al., 2017). The binding of NR to the hydrophobic surface of plastics causes them to fluoresce, which allows a more targeted analysis of the sample. A stock solution of 1 mg/mL NR in acetone was prepared and filtered (0.22 μm) into a clean glass (metal) screw-top vial. The solution was stored in the dark. A diluted working solution (10 μg/mL *n*-hexane) was prepared fresh on the day of use. Five millilitres of NR working solution was added to the sample filter. After 30 min of incubation, the NR solution was vacuum-filtered. The samples were stored in glass petri dishes in the dark at room temperature until analysis on the same or following day.

### 2.3 Visual observations

The NR-stained total atmospheric deposition samples were initially inspected with a fluorescence stereo microscope (Olympus SZX12; magnification 63x) and potential microplastics counted. All fibres in a sample were counted under bright field. Non-fibrous particles were observed under blue-violet light (ex.: 400–440, em.: 475 nm). Non-fibrous, fluorescing particles were counted as potential microplastics and marked with a pen on the filter if the following morphological inclusion criteria for microplastic identification also applied: (1) homogeneous material; (2) unnatural shape e.g. perfectly spherical; (3) shiny/glassy; (4) no cellular or organic structures visible; and (5) unnaturally coloured under bright-field compared to the rest of the sample (Hidalgo-Ruz et al., 2012). They were categorised morphologically as fragments (flattened and shard-like), films (transparent and thin (thinner than fragments)), granules (rounded) and foams (sponge-like texture). All potential non-fibrous microplastics in each sample were imaged and approximately sized using the software ImageJ v.1.5 (Schneider et al., 2012). For fibres, random fields of view were imaged and all fibres visible at that magnification were approximately sized using ImageJ until 10% of the total sample had been measured, due to their high abundance.

### 2.4 FTIR analysis

To determine the chemical composition of suspected microplastics, micro-Fourier-transform infrared (FTIR) spectroscopy was used. FTIR analysis was carried out with an infrared microscope (Perkin Elmer, USA) equipped with a mercury cadmium telluride (MCT) detector. A blank area of the edge of the filter was used as the background (64 scans). Due to their high abundance in atmospheric deposition and time constraints, 5% of fibres in each sample were randomly analysed (ranging from 7 to 15 depending on the sample density). All potential non-fibrous microplastics were analysed. Signals were obtained in reflectance mode and the spectral range was set from 500 to 4000 cm^−1^. The IR spectra were recorded (16 scans) with the Spectrum v5.3.1 software at a resolution of 4 cm^−1^. The resulting spectra were compared to a spectral library (Bio-Rad KnowItAll IR Spectral Library). Fibrous microplastics which consisted of purely petrochemical-based plastic were categorized as synthetic fibres. Particles with a hit quality index lower than 90% were categorized as “not-identified” due to ambiguity in interpretation of the match.

### 2.5 Measures to avoid contamination

Prior to use, all glassware was washed once with filtered 70% ethanol and three times with filtered (0.45 μm) ultrapure water (Purelab Ultra, ELGA Veolia, UK) by completely filling and emptying the glassware. All plastic components were removed from 1 L Duran bottles. The bottles were then filled with ultrapure water and placed in a sonicating bath for 5 min to remove any potential particles from the inside surface. The water was poured away, and the bottles cleaned with ultrapure water as outlined above. During sample collection, the researcher stood downwind of the sample to avoid contamination from clothing. All steps post-sample collection were conducted in the fume hood, although this was not under a laminar flow and was thus done in ‘off’ mode, to minimise exposure to the laboratory air. The 10% methanol solution used for extraction was pre-filtered (0.22 μm). All openings, including Duran bottles, filtration unit and beakers, were covered with aluminium foil to prevent airborne contamination. During screening of the samples, the microscope was covered with a plastic curtain to minimise deposition. Cotton laboratory coats were always worn. The glass petri dishes in which the samples were stored were cleaned with Kim wipes and 70% ethanol before use. A composite blank filter underwent all steps of the protocol, including 2 h under the microscope to emulate screening, to control for contamination. Just three cellulose fibres were observed in the blank.

### 2.6 Microplastic quantification

Based on the FTIR results, a correction factor was applied to estimate the number of microplastics present in atmospheric deposition samples using the known collection area and sampling duration:

$$Microplastics/m^{2}/d=\frac{Potential\;microplastics\times \%\;identified\times 31.85}{\mathrm{nd}}$$

where nd is the number of days sampling duration and the factor 31.85 standardises for a m^2^ area based on the sampling area 0.0314 m^2^. Expressing microplastic deposition as n/m^2^/d has been previously published and is a useful standard metric for geographical comparisons. This was calculated for both fibrous and non-fibrous microplastics.

### 2.7 Statistical analysis

The purpose of this study was to assess the microplastic profile in urban deposition. Previous studies have explored relationships between microplastic deposition and meteorological parameters. Simple correlations between the variability in microplastic abundance and meteorological parameters were therefore conducted in R Studio software (1.2 1335) (RStudio Team, 2015).

### 2.8 Local source areas and atmospheric transport

Bivariate polar plots (BPP) are a useful tool to identify possible source areas of an air pollutant near sample sites. BPP determine the mean value (or other metric) of an ambient pollutant concentration against wind direction and wind speed. Usually, highly time-resolved chemical and meteorological data (i.e. hourly) are merged for this. However, samples of microplastics were collected every 3–4 days. Constructing BPP using low-resolution data is made possible by copying the measured pollutant concentrations to the hourly meteorological data for the entire duration of individual samples, as presented in (Font et al., 2015). In this study, BPP were built using the concentrations of fibrous and non-fibrous microplastics and the hourly meteorological data collected at the sampling site. BPPs were calculated using the openair R-package (Carslaw and Ropkins, 2012).

Possible long-range transport of microplastic particles to the sampling location was estimated by first calculating the time that microplastics were suspended in the air; and second, by calculating the trajectory followed by the particles for that period. The time that microplastics were suspended in the air was calculated based on settling velocities for microplastics; and on typical boundary layer heights in London. Settling velocities were calculated following (Allen et al., 2019). However, where (Allen et al., 2019) calculations were based on a 25 µm dust particle, we do so for representative microplastic particles. Representative microplastics were deemed as follows: a non-fibrous microplastic (assumed spherical) with 100 µm diameter (the most frequent size class) and a material density of 1.05 g/cm^3^ (the most frequent non-fibrous polymer, polystyrene); and a fibrous microplastic for which an aerodynamic equivalent diameter was calculated for the most frequent diameter/length (20 µm/400 µm) with a material density of 1.184 g/cm^3^ (the most frequent fibrous polymer, polyacrylonitrile), following the equation by (Henn, 1996):

$${D_{a}\approx \left(d_{c}\ln2\beta \right)}^{1/2}D_{c}$$

where $D_{a}$ = aerodynamic equivalent diameter, $d_{c}$ = density, β = aspect ratio (length/diameter) and $D_{c}$ = cylindrical diameter. Settling velocity was calculated based on Stokes Law equation:

$$V_{t}=\frac{{\mathrm{gd}}^{2}\left(p_{p}-p_{m}\right)}{18\mu }$$

where g = gravity, d = particle diameter or V_t_ in the case of the fibrous microplastics, $p_{p}$ = density of particle, $p_{m}$ = density of medium and μ = viscosity of medium, where the medium is air. A boundary layer height of 350 and 750 m for night- and daytime, respectively, in London was assumed for Winter (Barlow et al., 2011). The resulting times that microplastics resided suspended in the air were 1 and 3-h for night- and daytime, respectively, for fibrous microplastics; and 1-h for non-fibrous microplastics.

The Hybrid Single-Particle Lagrangian Integrated Trajectory model (Hysplit) was then run using the Global Data Assimilation System (GDAS) gridded meteorological files at 0.5° spatial resolution (Rolph et al., 2017, Stein et al., 2015). The model was run in backward mode for the duration that microplastics were estimated to have been suspended in the air. The starting point of the backtrajectory was set at the sampling site at 100 m above ground level (magl).

## 3 Results

### 3.1 Urban atmospheric microplastic deposition

**Fig. 1:**
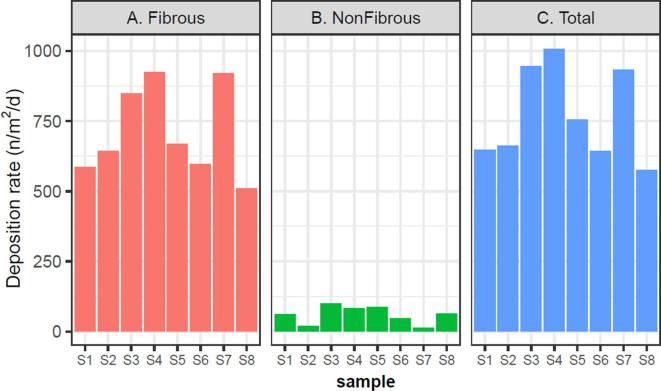
Time-series of deposition rates (n/m^2^/d) for (A) fibrous, (B) non-fibrous and (C) total microplastics.

Using Fourier-transform infrared microscopy (FTIR), microplastics were found in all deposition samples (Fig. 1A–C). This confirms that the lower atmospheric environment of urban London, an indicative megacity, is contaminated by microplastics and the polymer-shape-size configurations observed are removed from the atmosphere via deposition.

The deposition rate of fibrous microplastics was calculated to range from 510 to 925 fibrous microplastics/m^2^/d, with an average of 712 ± 162 microplastics/m^2^/d (mean ± SD) (Fig. 1A). The non-fibrous microplastic deposition rate ranged from 12 to 99 microplastics/m^2^/d, with an average of 59 ± 32 non-fibrous microplastics/m^2^/d (mean ± SD) (Fig. 1B). The average deposition rate of synthetic fibres and non-fibrous microplastics combined (total) was 771 ± 167 particles/m^2^/d (mean ± SD) (Fig. 1C). Fig. S2 shows along with the mean of different meteorological variables as the samples were collected. The sample-to-sample variability in the microplastic deposition rate did not correlate to any of the independent meteorological variables (R^2^ ranged from 0.0002 to 0.1517 for fibrous and 0.0016–0.1467 for non-fibrous microplastics).

### 3.2 The urban atmospheric microplastic profile

**Fig. 2:**
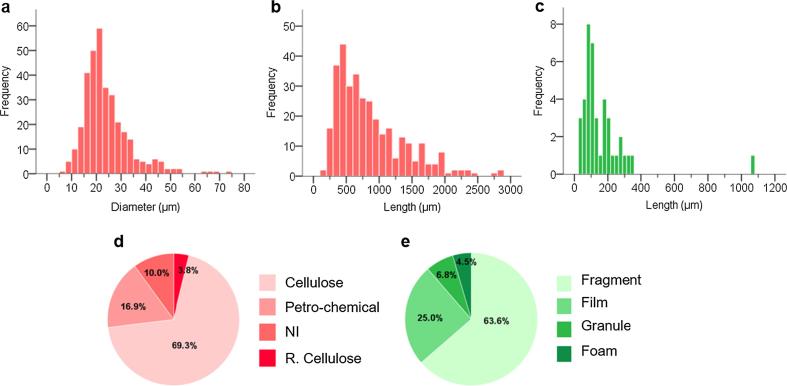
The profile of fibres and microplastics in total atmospheric deposition. (A) the size distribution of fibrous particle diameters (µm) based on 10% of fibres randomly intercepted on each sample filter; (B) the size distribution of fibrous particle lengths (µm) based on 10% of fibres randomly intercepted on each sample filter; (C) the size distribution of non-fibrous microplastic maximum dimensions (µm); (D) the proportional distribution of fibre materials; and (E) the proportional distribution of non-fibrous microplastic morphologies. NI = non-identifiable; R. Cellulose = regenerated cellulose.

Microplastics were approximately sized using Image J and classified by morphology. The modal diameter of the observed fibres was 20–25 μm (mean 24 ± 10 μm ± SD; Fig. 2A) with the thinnest and thickest being approximately 5 and 75 µm, respectively. The most abundant lengths were 400–500 μm (mean 905 ± 641 μm ± SD; Fig. 2B). Fibre frequency increased with decreasing length (Fig. 2B), suggesting the presence of shorter (<100 μm) fibres. The most abundant non-fibrous microplastics were between 75 μm and 100 μm in length (Fig. 2C). Except for one microplastic (low-density polyethylene (PE) film, 1080 μm), all non-fibrous microplastics were smaller than 350 μm. The smallest identified particle (high-density PE) was 25 μm and the average size of non-fibrous microplastics was 164 ± 167 μm (mean ± SD). Many fluorescent non-fibrous particles < 20 μm and smaller fibres were observed under the microscope but were not included due to the lower analytical threshold of the FTIR instrument employed (~20 μm). This could suggest that there are smaller microplastics in the air, which warrants further study.

**Fig. 3:**
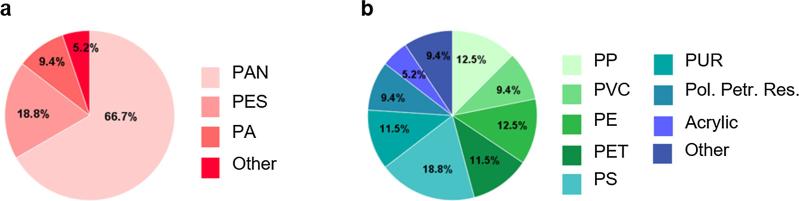
The composition of microplastics in total atmospheric deposition. (A) The proportional distribution of the identified petro-chemical-based fibrous microplastics; (B) the proportional distribution of the identified petro-chemical-based non-fibrous microplastics. PAN = polyacrylonitrile; PES = polyester, PA = polyamide; PP = polypropylene; PVC = polyvinylchloride; PE = polyethylene; PET = polyethylene terephthalate; PS = polystyrene; PUR = polyurethane; Pol. Petr. Res = polymerised petroleum resin.

Spectroscopic analysis found that 17% of fibres were synthetic (petro-chemical-based, Fig. 2D). Amongst these fibrous microplastics, polyacrylonitrile (PAN) was the most abundant polymer type (67%), followed by polyethylene terephthalate (PET (polyester); 19%) and PA (9%) (Fig. 3A). Five percent was categorized as ‘others’, which includes polymer types which were found no more than once across all samples such as polyurethane (PUR) and polypropylene (PP). Four percent of the total fibres were identified as regenerated cellulose, which refers to transformed natural polymers (e.g. rayon or acetate from cellulose). Most analysed fibres consisted of cellulose (69%), suggesting that cotton and other plant fibres, either from natural or anthropogenic sources, are the predominant fibre type in the air.

The most common non-fibrous morphology was fragments, accounting for 64%, followed by films (25%), granules (7%) and foams (4%) (Fig. 2E). Among the identified non-fibrous microplastics, eight kinds of synthetic polymers, namely polystyrene (PS), PP, PE, PET, PUR, polyvinylchloride (PVC), acrylic polymer and polymerized petroleum resin were identified (Fig. 3B). The highest proportion of non-fibrous microplastics was PS (including expanded polystyrene foams, i.e., Styrofoam), marginally so, comprising 19%. The category “other” included polyvinyl alcohol (PVA), styrene maleic anhydride, polynorbornene and vinyl ester resin. Examples of microplastics and their spectra are presented in Fig. S1.

### 3.3 Urban atmospheric microplastic source areas and transport

Settling velocities for representative microplastics, i.e. the most common size class-polymer type combination for fibrous and non-fibrous microplastics were calculated for the first time. Settling velocities of 0.32 m s^−1^ and 0.06 m s^−1^ were estimated for non-fibrous and fibrous microplastics, respectively. Assuming a wind speed of 5 m s^−1^, commonly observed throughout the study, the representative non-fibrous and fibrous microplastic particles would have travelled up to approximately 12 and 60 km, respectively.

**Fig. 4:**
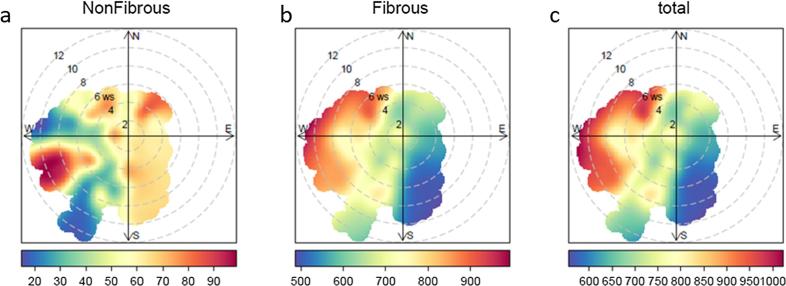
Bivariate polar plots of atmospheric microplastic deposition in central London for (A) non-fibrous, (B) fibrous and (C) total microplastics. The colour scales show microplastic deposition rate (n/m^2^/d); radial scales show wind speed (m/s), which increases from the centre of the plot radially out-wards (2 m s^−1^ increments).

Local source areas of microplastics were analysed using Bivariate Polar Plots (BPPs), which revealed differences for non-fibrous and fibrous microplastics (Fig. 4A–C). An increase in the deposition rates of non-fibrous microplastics was observed when wind was from the SW and to a lesser degree NE-E sectors, with deposition increasing with wind speed (Fig. 4A). There was also a clear increase of fibrous microplastic deposition when wind was from the SW – NW sector, with microplastic abundance increasing with wind speed (Fig. 4B). A difference in the source origin of fibrous and non-fibrous microplastics was indicated.

The long-range transport of microplastics to the measurement site was investigated by means of backtrajectory analysis (Figs. S3 and S4). The influence area of deposited microplastics in central London was centred in the urban area itself and south-east England. The influence area of fibrous microplastics was greater than that of non-fibrous microplastics, ranging from 640 to 8700 km^2^ for fibrous; and from 186 to 875 km^2^ for non-fibrous. This is most likely due to the differences in settling velocities assumed for representative microplastics of each morphology. There is no clear pattern in the origins of air masses and the variability of microplastic deposition rates between samples (see Figs. S3 and S4).

## 4 Discussion

The results from the present study corroborate findings in the few other existing studies, although the average atmospheric microplastics deposition rate is greater than what has previously been reported (Dris et al., 2016, Cai et al., 2017). Following (Allen et al., 2019), if only microplastics with lengths >200 µm are considered for the current study, deposition is approximately 718 microplastics/m^2^/d, (98% fibrous). This is considerably higher than for the confirmed microplastics in atmospheric deposition in China (26/m^2^/d), and in Paris (110/m^2^/d). It is almost 20 times higher than that observed in the French Pyrenees (40/m^2^/d). However, like atmospheric microplastics in the remote mountain catchment, most non-fibrous microplastics were <200 µm (78%). The deposition rate of non-fibrous microplastics (mean 59 ± 32 SD microplastics/m^2^/d) was comparable to other findings in Dongguan (36 ± 7 microplastics/m^2^/d) (Cai et al., 2017), but is substantially lower than what was found in the Pyrenees (322 m^2^/d) (Allen et al., 2019), probably due to the difference in analysed size ranges.

The large majority of microplastics observed in this (92%) and previous studies are fibrous and of similar lengths (Dris et al., 2016, Cai et al., 2017). Fibrous microplastics are postulated to derive from the wear of textiles, indicated by reported emissions via washing machine effluent (Napper and Thompson, 2016, Pirc et al., 2016). PAN comprised most fibrous microplastics. Often labelled as “acrylic”, PAN filaments are used to make knitted clothing such as socks, hats and sweaters. These samples were collected over winter. If fibrous microplastics are derived from textiles, e.g. upholstery, carpets and clothing, it can be predicted that abundance will be positively correlated with usage, occurring at greater levels in densely populated areas. Dongguan and London have comparative population sizes, with over 8 million inhabitants equivalent to approximately 3300 and 5100 inhabitants per km^2^, respectively. The city of Paris has a smaller (approximately 2 million inhabitants) but more concentrated (>21,600 inhabitants per km^2^) population. As similar levels of microplastics were found in Paris and Dongguan and higher levels measured in London, yet Paris has the greatest density of inhabitants in the city, population density alone does not seem to be the main influence on microplastic abundance. However, this does not take daytime population, from workers, tourists and visitors, into account. A SW – NW influence on fibrous microplastics observed in this study coincides with a commercial area which receives high footfall during the day. Additionally, PAN is used for outdoor textiles, with applications in tents, yacht sails and similar items, due to their high resistance to sun damage (Polymer Science Learning Centre, 2005) and as a reinforcer of cement. There were several construction sites east of the sampling site.

Little is known of the sources of airborne non-fibrous microplastics. In the present study, two geographical origins were suggested, but the heterogenous nature of the particles and applications of the observed polymers makes it difficult to discern local point-sources. It can be somewhat inferred through a combination of morphology and polymer type. Fragments and films were the most common shapes of non-fibrous microplastics. Fragments likely derive from thicker plastic products which can be recycled, films could originate from disposable, thin plastic items such as plastic bags and packaging and foam microplastics may be released from expanded polystyrene (EPS) items (Cai et al., 2017). PS is widely applied as thermal insulation and as packaging material in the fast food industry (Song et al., 2018). The second most common polymer types found - PE and PP (12% each) - are some of the most commonly produced plastic types and are also widely used as packaging material (Andrady, 2017). Hence, microplastics are likely being emitted from several sources, such as degrading exposed plastic in open landfill and the wider environment, or plastic waste which is abraded during waste transfer and processing activities. Granules accounted for 7% and resembled beads. They could represent primary microplastics, originating from accidental release at the production and transport stages. Polymerized petroleum resin accounted for 9% of non-fibrous microplastics. This polymer is used together with other kinds of resins in rubber tyres, road paint and in the production of varnish and construction paints (Zohuriaan-Mehr and Omidian, 2000). Tyre wear is a recognised component of particulate air pollution (Panko et al., 2019) and potentially atmospheric deposition (Bergmann et al., 2019). Whilst the observation of polymerised petroleum resin particles may indicate tyre wear, it was beyond the scope of the current study to include tyre wear; the visual screening of fluorescing, non-fibrous particles used traditional morphological criteria, which do not include tyre wear characteristics, to discriminate potential microplastics. However, given the substantial microplastic environmental loadings predicted form tyre wear (Kole et al., 2017), it is recommended that future studies adapt existing methods to include these particles. Despite using both Nile Red staining and morphological criteria, 33% of suspected non-fibrous microplastics were also cellulose based, highlighting the importance of compositional analysis even when staining.

Microplastics likely become airborne and transported via wind deflation. In the current study, estimated transport was less than that estimated for a remote pristine area (Allen et al., 2019), which suggested microplastics had travelled up to 95 km. However, different assumptions (size/aerodynamic equivalent diameter and density) were made about the particles and the contrasting environments likely receive different meteorological conditions. Furthermore, the results of the current and other studies (Allen et al., 2019) should be interpreted with caution given the small sample size, and further research is encouraged to build this evidence base.

The lack of significant correlations with meteorological variables implies that local sources have a greater influence on microplastic deposition in central London. This contrasts to the findings of (Allen et al., 2019). Thus, meteorological parameters may be important for remote locations away from sources, but less so for central urban areas, which are likely to be a source of emissions to the wider environment. Future studies should aim to monitor specific microplastic sources and characterize their composition to footprint ambient concentrations. Combining chemical composition and atmospheric dispersion could enhance identification and quantification of the intensity of the sources to ambient concentrations and therefore facilitate regulation and prevent wider environmental contamination.

Evidence on the permeation of microplastics into the atmospheric environment is building. Once airborne, they could remain suspended for days or weeks before being removed via precipitation; giant mineral dust particles are transported thousands of kilometres from their sources (van der Does et al., 2018). Thus, the atmosphere represents a diffuse source of microplastics, which may deposit into different environments, including oceans, where smaller microplastics may be re-aerosolised via wave breaks and bursting bubbles, and again transported. The question that comes to mind is, are we dealing with a ‘global microplastics cycle’? With regards to human health, exposure is still unclear. Should microplastics observed in the present study be inhaled, they are likely to rapidly deposit in the upper airway (nose, mouth, throat) and be swallowed, leading to exposure in the gut. Many fluorescent non-fibrous particles < 20 μm were observed under the microscope but were not included in the results due to the lower analytical threshold of the FTIR instrument employed (~20 μm). This could suggest that there are smaller microplastics in the thoracic and potentially respirable size ranges present in the air and warrants further study; hence, airborne pathways should be considered in future assessments of daily intake via both air and diet.

## 5 Conclusion

Here we report on the first evidence of microplastic deposition in urban London, indicative of a highly populated European city. Microplastics were found in every sample and average deposition rates were greater than what has previously been reported. It has been suggested that precipitation may influence microplastic deposition, however, there was no observed influence of meteorological parameters in the present study. This suggests cities are a source of airborne microplastics to the wider environment. In order to minimise microplastic emissions, key contributors need to be identified. Polymer type alone is not enough of a trace. Hence, studies which identify point-sources of emissions are needed. Little is known of the dynamics of atmospheric microplastic dispersion and therefore fate, and it is recommended that future studies attempt to elucidate these in order to mitigate potential impacts.

Consolidating our understanding of human exposure is a pressing issue. The associated health effects of particulate matter (PM) are well-established and can occur following occupational exposures, e.g., to mineral fibres, but are primarily associated with road transport and fuel burning emissions. As global pressure to reduce such emissions increases, PM composition is likely to shift. In combination with a predicted increase in plastic use, especially in the textile sector (4%/year), the proportional concentration of airborne microplastics will become increasingly important. It is therefore timely to establish baseline knowledge of global airborne microplastic burdens and begin to understand what their potential role in PM-associated health effects might be. However, if the field is to progress, higher throughput, resolved instrumentation, scientific accuracy and robust reporting is necessary.

## CRediT authorship contribution statement

**S.L. Wright:** Conceptualization, Methodology, Formal analysis, Writing - review & editing, Visualization, Supervision, Project administration. **J. Ulke:** Methodology, Validation, Formal analysis, Writing - original draft, Writing - review & editing, Funding acquisition. **A. Font:** Software, Formal analysis, Writing - review & editing, Visualization. **K.L.A. Chan:** Resources, Writing - review & editing. **F.J. Kelly:** Writing - review & editing, Supervision.

## Declaration of Competing Interest

The authors declare that they have no known competing financial interests or personal relationships that could have appeared to influence the work reported in this paper.
