# Supplementary material for: Atmospheric microplastic deposition in an urban environment and an evaluation of transport
Source: Environ Int. 2020 Mar;136:105411. doi: 10.1016/j.envint.2019.105411 (PMC7013824; doi:10.1016/j.envint.2019.105411)
Supplement: Supplementary data 1 [file mmc1.docx]

Supplementary Material

Atmospheric microplastic deposition in an urban environment and an evaluation of transport

S. L. Wright ^1‡*^, J. Ulke ^1, †‡^, A. Font ^1, 2^, K. L. A. Chan ^3^ and F. J. Kelly ^1, 2^

^1^ MRC Centre for Environment and Health, Analytical, Environmental and Forensic Sciences, King’s College London, London, United Kingdom

^2^ Environmental Research Group, Analytical, Environmental and Forensic Sciences, King’s College London, London, United Kingdom

^3^ Institute of Pharmaceutical Science, School of Cancer and Pharmaceutical Sciences, King’s College London, London, United Kingdom

^†^ Permanent Address: Institute of Nutritional Science, University of Potsdam, Nuthetal, Germany

^‡^ joint first author

^*^ corresponding author stephanie.wright@kcl.ac.uk

**Supplementary Materials**

**
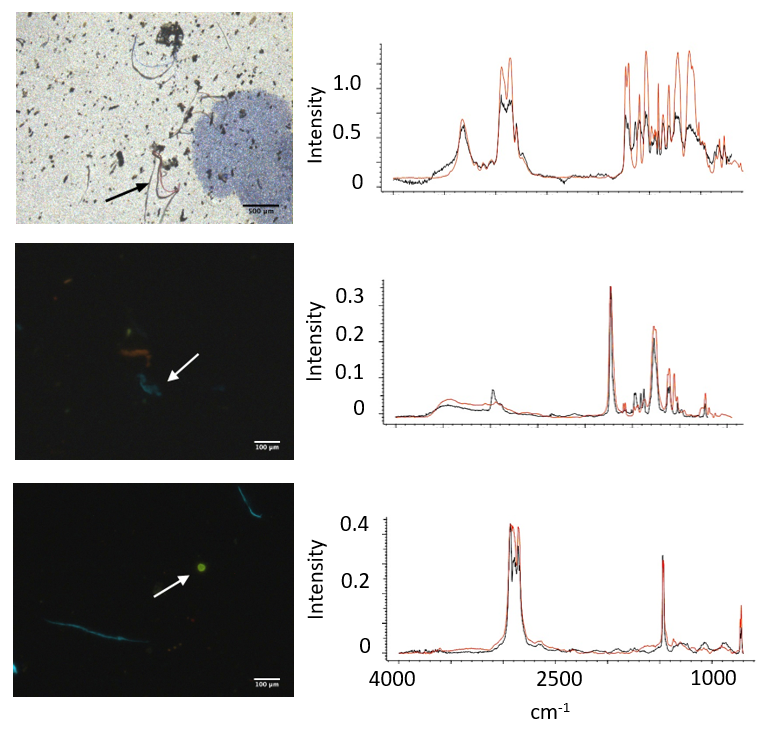
**

Figure S 1. Examples of fibrous and non-fibrous microplastics observed in samples and their corresponding FTIR spectra (black) and reference spectra (red) for (top to bottom) polyurethane (PUR, 94% match), polyethylene terephthalate (91% match) and high-density polyethylene (97% match) [scale bar = 500 µm (top) and 100 µm (middle, bottom)].


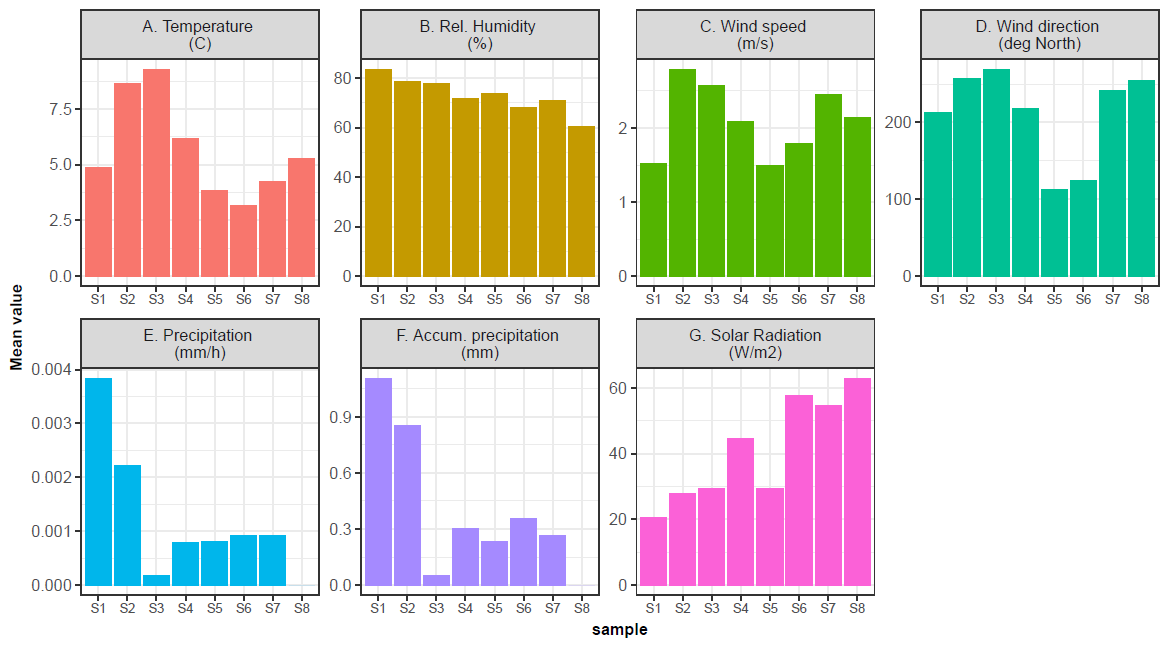


Figure S2. Time series of the meteorological variables during the sampling campaign.


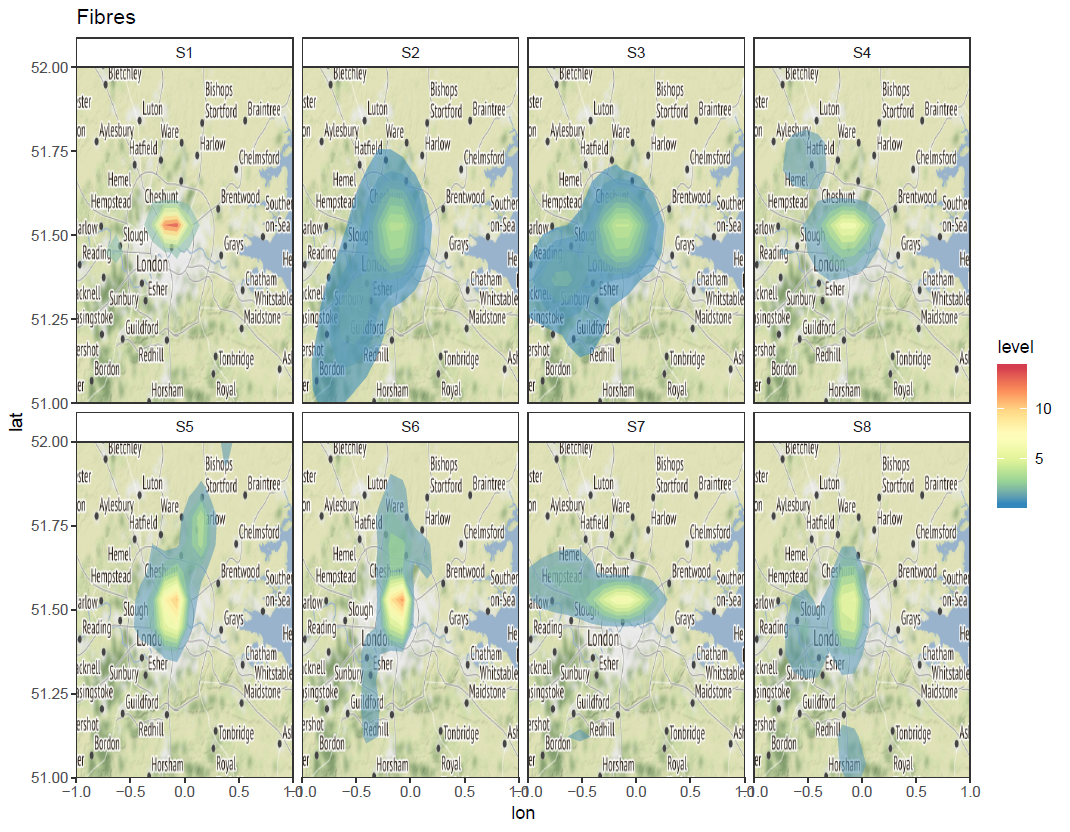


Figure S3. Influence area for fibrous microplastic samples as calculated by the backtrajectory model. Level indicates the number of air parcels originating in that area.


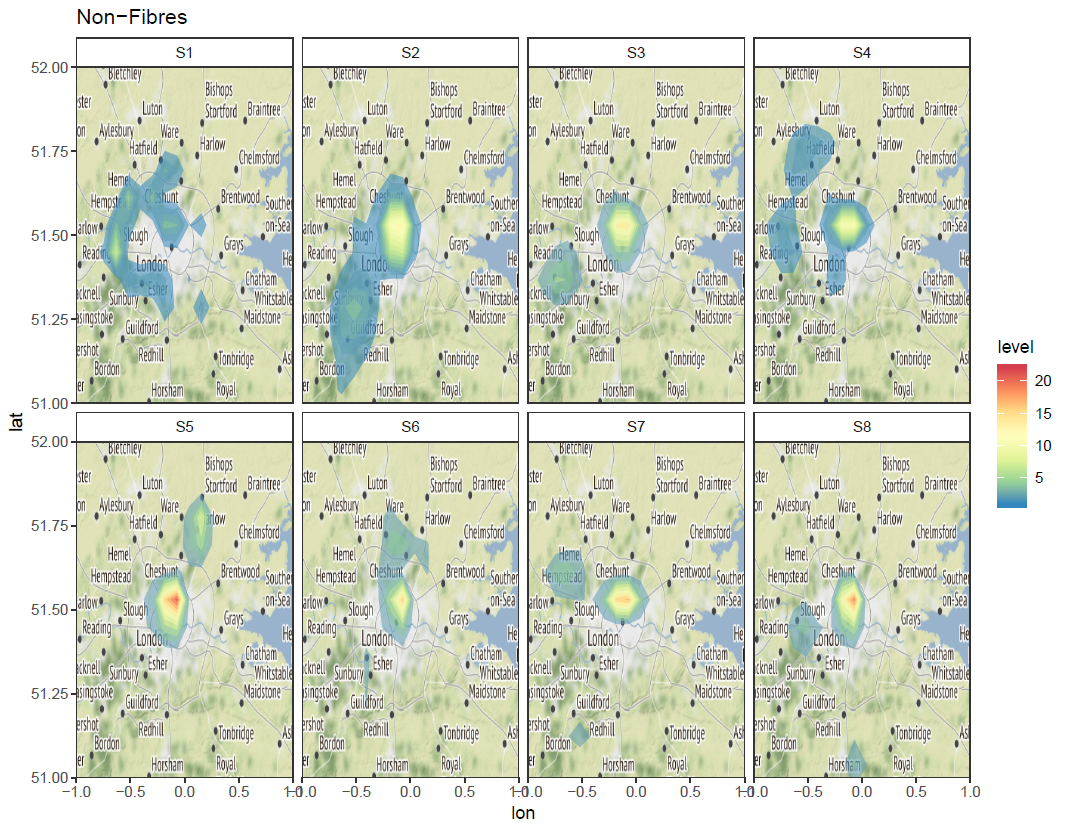


Figure S4. Influence area for non-fibrous microplastic samples as calculated by the backtrajectory model. Level indicates the number of air parcels originating in that area.
